# Long-Term Viruria in Zika Virus–Infected Pregnant Women, Brazil, 2016

**DOI:** 10.3201/eid2311.170078

**Published:** 2017-11

**Authors:** Ana Carolina B. Terzian, Cássia Fernanda Estofolete, Rafael Alves da Silva, Denise Cristina Mós Vaz-Oliani, Antonio Hélio Oliani, Cinara Cássia Brandão de Mattos, Luiz Carlos de Mattos, Paula Rahal, Maurício L. Nogueira

**Affiliations:** São José do Rio Preto School of Medicine, São José do Rio Preto, Brazil (A.C.B. Terzian, C.F. Estofolete, R.A. da Silva, M.L. Nogueira);; São José do Rio Preto School of Medicine Foundation, São José do Rio Preto (D.C.M. Vaz-Oliani, A.H. Oliani, C.C.B. de Mattos, L.C. de Mattos);; São Paulo State University, São José do Rio Preto (P. Rahal)

**Keywords:** Zika, pregnancy, viruria, vector-borne infections, viruses, Zika virus, urine, flavivirus, Brazil, pregnant women, newborns, outcomes in newborns

## Abstract

During the 2016 Zika virus outbreak in Brazil, we detected Zika virus RNA in urine samples collected from Zika virus–positive pregnant women during different stages of pregnancy. Women had positive and negative intervals of viruria; 3 newborns had adverse outcomes. Further research is needed to clarify the relationship between viruria and outcomes for newborns.

Zika virus is a reemerging flavivirus that was first isolated in Uganda in 1947 during the course of a yellow fever virus survey (*1*). The virus remained at low transmission levels until the first Zika virus outbreak in Micronesia in 2007, spreading to the Pacific Islands in 2013–2014 and reaching Brazil in 2015 (*2*). Before the outbreak in Brazil, Zika virus infection was considered a mild febrile illness that did not produce severe outcomes. However, the cases of microcephaly, fetal abnormalities, and Guillain–Barré syndrome reported in Brazil reshaped our knowledge of the course of infection with this flavivirus, and demand for a rapid molecular approach to diagnose Zika virus infection began (*2*).

Molecular diagnosis of flavivirus infections is usually performed with blood or serum samples during the viremic period, which is sustained for ≈5–7 days (*3*). In the case of Zika virus infection, urine, saliva, and semen have been identified as additional sources that can be used for viral RNA detection. Urine and semen are especially useful; detection in these fluids is prolonged, being present even after the clearance of viremia (*4*,*5*). We describe the detection of Zika virus RNA in the urine from a group of Zika virus–positive pregnant women.

## The Study

During the February–October 2016 Zika virus outbreak, pregnant patients with Zika-like symptoms were treated at the Public Health Authority in São José do Rio Preto, Brazil. We collected serum and urine samples from these women during their first visit to the facility after symptom onset (Table) and tested their samples for Zika virus RNA (*6*). We extracted viral RNA from 140 µL of each sample using the QIAamp Viral RNA Mini Kit (QIAGEN, Valencia, CA, USA) according to the manufacturer’s instructions. We performed a 1-step, quantitative, real-time, fluorescent probe–based PCR with primers targeting the Zika virus envelope gene as previously described (*6*).

**Table:** Long-term viruria in 13 Zika virus–positive pregnant women, São José do Rio Preto, Brazil, 2016*

Pt. no.	Week of gestation at virus detection	Days until first collection	Sample no., week of gestation/sample type and result/C_t_									Week of gestation at birth	Adverse newborn outcome
1	2	3	4	5	6	7	8	9
1	16	2	16/S+/ 29.34; 16/U+/ 31.31	17/U+/33	19/U–	22/U+/38.01	24/U–	27/U–	31/U–	36/U+/37.24		38	No
2	4	3	4/S+/ 35.62; 4/U+/ 36.39	6/U–	10/U–	12/U–	17/U–	23/U+/38.21	28/U+/37.63	32/U+/37.58	36/U–	39	NA
3	30	5	30/S+/ 37.33; 30/U+/ 25.08	32/U–	34/U+/35.79	37/U+/36.42						38	No
4	17	4	17/S+/33	18/U–	23/U–	28/U+/38.06	32/U+/37.82	34/U–	36/U+/37.03			39	No
5	27	4	29/S+/ 37.35	31/U+/36.86	33/U–	35/U–						36	Unilateral abnormal OAE
6	14	1	14/S+/ 33.51; 14/U+/ 32.75	14/S–; 15/U+/37.72	21/U–	27/U–	31/U+/36.59	35/U+/36.98				38	No
7	14	39	20/U+/ 38.5	22/U–	27/U+/34.88	31/U–	36/U–					39	No
8	15	6	16/S+/ 37.28; 16/U+/ 34.56	18/U–	23/U+/36.6	27/U–	32/U+/32.68	36/U+/36.64				39	No
9	17	16	18/U–	22/U+/37.58	27/U–	32/U+/37.62	38/S–					38	No
10	21	32	26/U–	28/U–	32/U–	35/U+/ 38.1						37	Subependymal cysts
11	5	12	6/S+/ 37.85	14/U–	19/U–	23/U–	27/U+/37.71	31/U–	35/U–			38	No
12	19	47	25/U+/ 36.87; 25/S–	30/U+/36.81	32/U+/38.2	36/U–						38	No
13	24	41	25/U–	27/U–	31/U+/38.15	36/U+/33.9						37	Unilateral abnormal OAE

*Plus signs (+) indicate positive test results (C_t_ <38.5) and minus signs (–) indicate negative test results (C_t_ >38.5 or undetermined) (*6*). All pregnant women initially tested positive for Zika virus RNA. For some women (patient nos. 7, 8, 9, 10, 12, 13), data from the initial samples were not available. C_t_, cycle threshold; NA, not available; OAE, otoacoustic emissions; pt., patient; S, serum; U, urine.

Pregnant women who were Zika virus positive by quantitative reverse transcription PCR (RT-qPCR) (*6*) were monitored by a multidisciplinary medical team at the Criança e Maternidade Teaching Hospital in São José do Rio Preto, and viruria levels in urine were measured until delivery. RT-qPCR results were considered positive if the cycle threshold (C_t_) was <38.5 and negative if the C_t_ was >38.5, as described previously (*6*). The pregnant women considered for enrollment in this study were 4–38 weeks into their pregnancies. This work was approved by the institutional review board of the São José do Rio Preto School of Medicine.

RT-qPCR results showed that urine samples taken from patients at different weeks of gestation were positive for Zika virus RNA; however, some of the consecutively collected samples were negative, showing positive and negative intervals (Table). Although not all samples were positive, we detected viral RNA in urine samples acquired sequentially over a course of >4 weeks for 6 of 13 patients (nos. 2, 4, 6, 8, 12, 13). The maximum period of detection was 7 months (patient no. 2). The number of collections varied depending on when during the pregnancy the patient started treatment at the facility and when the last urine sample was collected before delivery. Of the 12 newborns for which outcomes were known, 3 (25%) had adverse outcomes: 1 had subependymal cysts and 2 had unilateral abnormal otoacoustic emissions (Table).

## Conclusions

We report findings of long-term viruria among Zika virus‒positive pregnant women, with Zika virus RNA being detectable in urine during different stages of pregnancy. Plasma and serum are considered the biologic fluids of choice for the molecular diagnosis of Zika virus for <5 days after illness onset (*6*,*7*). However, after this short viremic period, these fluids are no longer ideal for diagnosis, although whole blood has been found to be more sensitive than plasma for Zika virus detection (*6*–*8*). A diagnosis on the basis of viremia is not recommended for cases in pregnant women >1 week after the onset of symptoms (*7*). In this study, Zika virus RNA was detected in the urine samples from pregnant women collected 1‒7 months after the onset of symptoms; however, viruria was not detected after the women delivered. We suspect that this phenomenon might occur because virus replication is maintained in fetal tissues (e.g., brain, placenta, umbilical cord, liver, lung, spleen, muscles) at different viral loads (*7*,*9*) and that these tissues, therefore, act as virus reservoirs (*9*). The intermittence of Zika virus RNA detection in urine samples (fluctuation between negative and positive C_t_ results) was likely due to the sensitivity of the test. More studies are necessary to understand the dynamics of the replication of Zika virus in fetal tissues and its connection to viruria in pregnant women.

Urine, semen, and saliva have been described as specimens that can be used in the molecular detection of Zika virus after the clearance of viremia (*4*,*5*). The detection of RNA of other flaviviruses in urine and semen samples has been previously described (*5*,*10*), and for these viruses, viral RNA is detectable for longer durations and at higher viral loads in semen and urine samples than they are in plasma samples (*5*,*11*). The presence of Zika virus RNA in semen samples supports the theory of sexual transmission and has led to discussions of Zika virus replication in the genitourinary tract (*5*). This hypothesis is supported by West Nile virus literature showing the detection of virus RNA in the renal cells of experimentally infected hamsters (*10*). Zika virus viruria and the presence of the virus in the peritubular cells of the testes (*12*,*13*) suggests that renal tissue might be a repository of the virus. However, in our study, Zika virus RNA was not detected in urine samples at all stages of pregnancy.

The use of urine for diagnosis represents an additional tool for virus detection. Urine is easy to obtain from pregnant women (because collection of this sample does not induce physiologic stress) and can be taken >5 days after the onset of clinical symptoms (*14*,*15*). The detection of Zika virus RNA (particularly in blood, urine, and semen) >5‒7 days after symptom onset is a new feature in the body of knowledge available on flaviviruses. The spread of Zika virus and its consequences have led to a paradigm shift and a race to develop new technologies to help understand the virus and prevent infection.

Considering that some mothers had viruria just before delivery and newborns without adverse outcomes, the adverse outcomes of the 3 newborns might or might not have been related to the viruria. Because microcephaly and fetal abnormalities have been attributed to Zika virus, continuing to monitor women who had Zika virus infections during their pregnancies will be essential to manage adverse fetal outcomes. The monitoring of Zika virus viruria in pregnant women through the regular collection of urine samples over the course of the pregnancy proved to be a workable approach; however, the meaning of viruria and the consequences for newborns will need to be evaluated further. Whether virus replicates in fetal tissues and uses them as reservoirs remains to be determined.
